# Similarities and differences between rat and mouse chondrocyte gene expression induced by IL-1β

**DOI:** 10.1186/s13018-021-02889-2

**Published:** 2022-02-04

**Authors:** Dao-Fang Ding, Yan Xue, Jun-Peng Zhang, Zeng-Qiao Zhang, Wen-Yao Li, Yue-Long Cao, Jian-Guang Xu

**Affiliations:** 1 School of Rehabilitation Science, Shanghai University of Traditional Chinese Medicine, Shanghai, 201203 China; 2 Center of Rehabilitation Medicine, Yueyang Hospital, Shanghai University of Traditional Chinese Medicine, Shanghai, 200437 China; 3 Shanghai Yangzhi Rehabilitation Hospital (Shanghai Sunshine Rehabilitation Centre), Tongji University School of Medicine, Shanghai, 201613 China; 4 Shi’s Center of Orthopedics and Traumatology, Shuguang Hospital, Shanghai University of Traditional Chinese Medicine, Shanghai, 201203 China

**Keywords:** Osteoarthritis, Chondrocyte inflammatory model, RNA-seq, Differentially expressed gene, Proliferation

## Abstract

**Background:**

Osteoarthritis (OA) is the most prevalent degenerative joint disease. In vitro experiments are an intuitive method used to investigate its early pathogenesis. Chondrocyte inflammation models in rats and mice are often used as in vitro models of OA. However, similarities and differences between them in the early stages of inflammation have not been reported.

**Objective:**

This paper seeks to compare the chondrocyte phenotype of rats and mice in the early inflammatory state and identify chondrocytes suitable for the study of early OA.

**Methods:**

Under similar conditions, chondrocytes from rats and mice were stimulated using the same IL-1β concentration for a short period of time. The phenotypic changes of chondrocytes were observed under a microscope. The treated chondrocytes were subjected to RNA-seq to identify similarities and differences in gene expression. Chondrocytes were labelled with EdU for proliferation analysis. Cell proliferation-associated proteins, including minichromosome maintenance 2 (MCM2), minichromosome maintenance 5 (MCM5), Lamin B1, proliferating cell nuclear antigen (PCNA), and Cyclin D1, were analysed by immunocytochemical staining, cell immunofluorescence, and Western blots to verify the RNA-seq results.

**Results:**

RNA-seq revealed that the expression patterns of cytokines, chemokines, matrix metalloproteinases, and collagen were similar between the rat and mouse chondrocyte inflammation models. Nonetheless, the expression of proliferation-related genes showed the opposite pattern. The RNA-seq results were further verified by subsequent experiments. The expression levels of MCM2, MCM5, Lamin B1, PCNA, and Cyclin D1 were significantly upregulated in rat chondrocytes (*P* < 0.05) and mouse chondrocytes (*P* < 0.05).

**Conclusions:**

Based on the findings, the rat chondrocyte inflammation model may help in the study of the early pathological mechanism of OA.

**Supplementary Information:**

The online version contains supplementary material available at 10.1186/s13018-021-02889-2.

## Background

Osteoarthritis (OA) is a common chronic disease-causing type of articular cartilage degeneration. The World Health Organization approximates that 9.6% of men and 18% of women over 60 years of age are in the early stages of OA pathogenesis [1, 2]. China has a high OA prevalence, and its burden is substantial [3, 4]. OA progression is clinically slow and unpredictable, and related clinical studies focus on the middle and late stages of the disease. As a result, these findings may not reflect the molecular events and structural changes of the joints in an accurate and timely manner [5, 6]. Early OA pathogenesis is the most vital stage but is often ignored and not fully understood [7, 8]. Since it is relatively difficult to obtain clinical samples, animal models of OA are often used for alternative research [9]. Nevertheless, animal experiments are time-consuming with many uncontrollable factors [10].

In contrast with the more complex in vivo experiments, in vitro experiments are direct and convenient in studying early disease pathogenesis [11]. To better explore the pathological mechanism of early OA, we searched the literature related to in vitro experiments on OA. Rat and mouse chondrocytes were more often selected as study subjects than human chondrocytes (Additional file 4: Fig. S1). Moreover, several studies have used IL-1β to simulate the OA environment in chondrocytes to assess the pathological mechanism of OA [12, 13]. The chondrocytes were from either rats or mice, and the intervention conditions were similar. Because of the species differences between rats and mice, we sought to identify whether chondrocytes from different species exhibit different performances after induction with similar IL-1β concentrations for a short period of time.

This work aims to compare and confirm similarities and differences in gene expression between rat and mouse chondrocytes under similar inflammatory induction conditions. This report is geared towards a simple and appropriate in vitro inflammatory model for the study of the early pathological mechanism of OA.

## Methods

### Animals

Sprague–Dawley (SD) rats and C57BL/6 mice were purchased from Shanghai Xipur-Bikai Experimental Animal Co., Ltd. (Shanghai, China). All the experimental animals were sacrificed through decapitation after inhaling diethyl ether.

### Isolation, culture, and intervention of chondrocytes

The primary culture of chondrocytes was performed according to the published literature [14]. Chondrocytes were isolated from the articular cartilage of newborn rats and mice (24 h old) and then dispersed in 0.1% collagenase type II (C6885, Sigma-Aldrich, Switzerland) in a shaker at 37 °C for 3 h. The chondrocytes were collected and cultured in Dulbecco’s modified Eagle’s medium (DMEM, Biowest, France) supplemented with 10% FBS (Biowest, France) and 1% penicillin–streptomycin. Chondrocytes at passage 1 were used in all experiments to prevent chondrocyte dedifferentiation during long-term in vitro expansion [15].

The chondrocytes of rats and mice were divided into the control group (RC = rat control group, MC = mouse control group) and model group (RM = rat model group, MM = mouse model group). The chondrocytes in the model groups were stimulated with 20 ng/ml IL-1β (R&D Company) for 24 h and extracted for sequencing.

### RNA-seq and data analysis

RNA samples for sequencing were extracted from three independent samples of each group to reduce individual differences. A total amount of 1.5 µg RNA extract was used, and cDNA library construction and RNA-seq (Illumina HiSeq 4000) were performed by Shanghai Life Genes Technology Co., Ltd. An RNA integrity number (RIN) > 8 confirmed the integrity of the RNA. RNA integrity was evaluated using the RNA Nano 6000 Assay Kit of the Bioanalyzer 2100 system (Agilent Technologies, CA, USA). Sequencing libraries were generated using the NEBNext® Ultra™ RNA Library Prep Kit for Illumina® (NEB, USA). For selection of appropriately sized cDNA fragments, the library fragments were purified using the AMPure XP system (Beckman Coulter, Beverly, USA). Finally, library quality was assessed on the Agilent Bioanalyzer 2100 system. After sequencing, the data from different groups were analysed.

The network of common genes and the KEGG pathways were drawn using Cytoscape 3.6.1. A sample with a fold change greater than 0.85, P value less than 0.05, and at least one fragment per kilobase million (FPKM) ≥ 1 (excluding low expression genes) was considered an effective differentially expressed gene (DEG). DEGs were also annotated in GO and KEGG databases to detect gene functions and pathways. Notably, high-throughput sequencing data are available through the GEO database with the access number GSE163080.

### EdU (5-ethynyl-2′-deoxyuridine) assay

Chondrocytes of each group were seeded in 6-well plates at a density of 3 × 10^5^ cells/well. The proliferative capability of the chondrocytes was detected by a BeyoClick™ EdU Cell Proliferation Kit with DAB (Beyotime, Shanghai, China). The experimental process strictly followed the manufacturer’s instructions. The nuclei were detected using DAPI staining solution (Cat. No. A1001, Aleichem, Germany). After the cells were washed in PBS, they were observed under an inverted microscope (Olympus IX73, Tokyo, Japan). Three technical repeated experiments were conducted in each group.

### Immunocytochemistry staining

Chondrocytes of each group were seeded in 6-well plates at a density of 3 × 10^5^ cells/well, fixed on slides with 4% paraformaldehyde at room temperature for 30 min, and then rinsed using phosphate buffer (PBS). The cells were incubated using 0.1% Triton X-100 for 20 min, followed by PBS rinsing. Chondrocytes were incubated with 5% bovine serum albumin at 37 °C for 20 min and then incubated overnight with primary antibody against MCM2 (A5172, Bimake, China) at 4 °C. The cells were then washed with PBS three times and incubated with a secondary antibody in 1% BSA for 30 min at RT. Diaminobenzidine (DAB) was used as a substrate for colour development. The nuclei were stained using 10 ng/ml DAPI (Cat. No. A1001, Aleichem, Germany) to mark all cells; they were observed under an inverted microscope.

### Cell immunofluorescence

Chondrocytes were seeded onto coverslips and maintained in complete DMEM medium or complete DMEM medium plus IL-1β for 24 h. The culture medium was discarded, and the coverslips were rinsed twice with PBS pH 7.4. The cells were fixed with 4% paraformaldehyde, washed three times using PBS, permeabilized with 0.1% Triton X-100 in PBS for 30 min at room temperature (RT), and washed with PBS three times. Thereafter, the cells were incubated in PBST with 10% BSA for 60 min to block nonspecific antibody binding, incubated with primary MCM5 antibody (A5489, Bimake, China) in 1% BSA for 60 min at RT, washed with PBS three times, and incubated with a secondary antibody in 1% BSA for 30 min at RT. The nuclei were stained using 10 ng/ml DAPI (Cat. No. A1001, Aleichem, Germany).

### Western blot

Chondrocytes were collected in lysis buffer (Beyotime, P0013B) containing protease inhibitor (PMSF). The lysates were centrifuged at 12,000×*g* at 4 °C for 10 min. Protein concentrations were measured using a BCA Protein Assay Kit (Cat. No. 23227, Pierce, USA). SDS-PAGE (15%) was used to separate the proteins and transferred to PVDF membranes based on a standard protocol. These membranes were incubated with primary antibodies and probed with the corresponding secondary antibodies. Protein visualization was conducted using enhanced chemiluminescence (Pierce Biotechnology, Rockford, USA). The following antibodies were utilized: PCNA (A5324, Bimake, China), Cyclin D1 (A5035, Bimake, China), and GAPDH (#2118, CST, USA).

### Statistical analysis

Statistical analyses were performed using SPSS 20.0 software (IBM SPSS, Inc., Chicago, IL, USA). The data are expressed as the mean ± standard deviation. Data between the two groups were compared using an unpaired t test. All data are normally distributed. When the sample size was 3, the sample power was 0.9832. Cohen's D statistic was used to test the effect size (ES), and the ES value was 4.47. GraphPad Prism 8.0 (GraphPad Software San Diego, CA) was applied for data drawing. *P* < 0.05 was considered statistically significant.

## Results

### The expression of common genes and KEGG pathways in rat and mouse chondrocytes by enrichment analysis

**Fig. 1:**
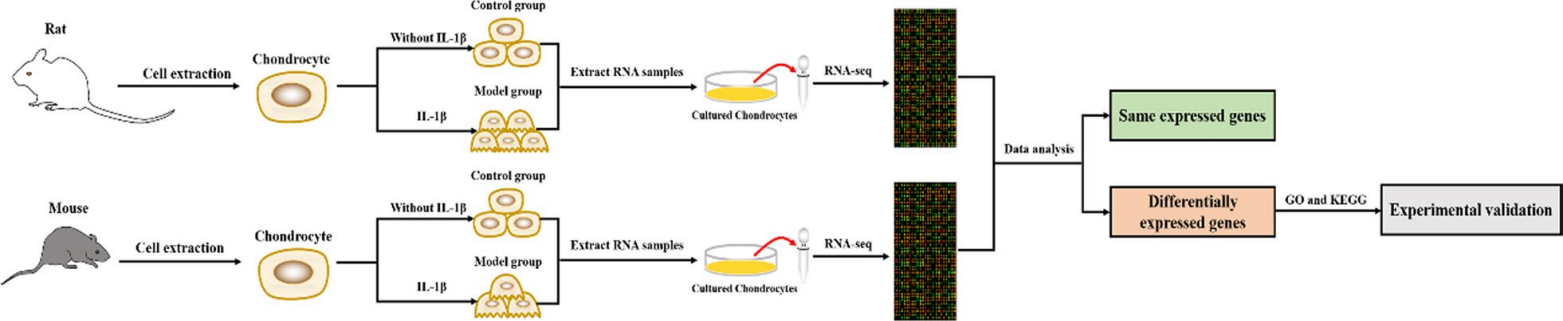
Overview of this study

**Fig. 2:**
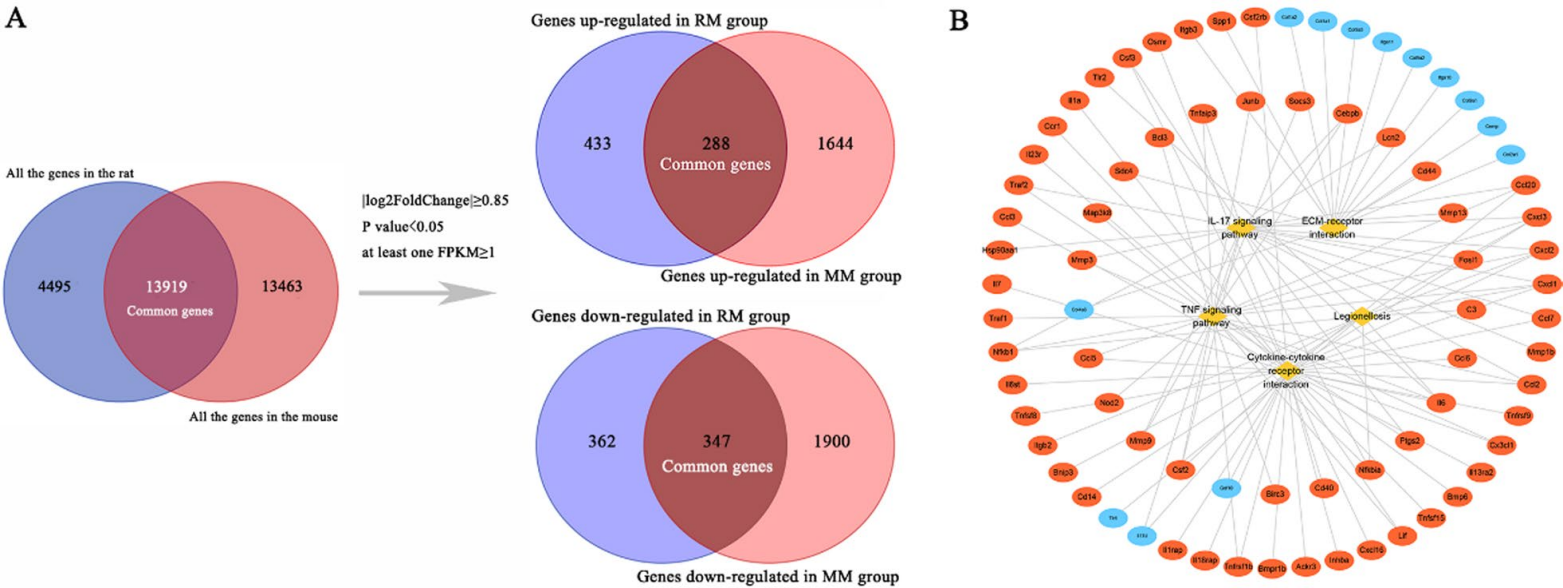
Common gene and pathway analysis. **A** Flowchart of screening common genes. **B** Target-pathway network. The yellow rhombus nodes represent the pathways, the red ellipse nodes represent the genes with high expression, and the blue ellipse nodes represent the genes with low expression

The methodology of this study is summarized in Fig. 1. After sequencing, 13,919 genes were shared by rats and mice. Of these, 721 genes had significantly upregulated expression in the RM group, while 1932 genes had significantly upregulated expression in the MM group. Moreover, 709 genes had significantly downregulated expression in the RM group, and 2247 genes w had significantly downregulated expression in the MM group. By taking the intersection of genes, we found that 288 genes had upregulated expression, while 347 genes had downregulated expression in both the rat and mouse model groups. Notably, 635 genes showed a similar expression pattern (Fig. 2A; Additional file 1: Table S1). Through KEGG pathway enrichment analysis, 5 enriched signalling pathways were selected and sorted by P value: the TNF signalling pathway, IL-17 signalling pathway, cytokine-cytokine receptor interaction, and other signalling pathways (Table 1). Among the 5 signalling pathways, 76 genes, including cytokines (IL-1, IL-6, IL-13, and IL-18), chemokines (CXCL1, CXCL2, CXCL3, and CXCL16), matrix metalloproteinases (MMP3, MMP9, and MMP13), and collagen (Col1a1, Col1a2, Col2a1, Col9a1, Col9a2, and Col9a3), were primarily involved (Fig. 2B). In both the rat and mouse model groups, the expression of cytokines, chemokines, and matrix metalloproteinases was upregulated, whereas the expression of collagen was downregulated.

**Table 1:** Information about related pathways (sorted by *P* value)

Pathway ID	Pathway name	Number of genes	*P* value (RM group)	*Q* value (MM group)
ko04668	TNF signalling pathway	26	1.63E−14	8.06E−14
ko04657	IL-17 signalling pathway	23	3.06E−13	3.17E−13
ko04060	Cytokine-cytokine receptor interaction	36	1.52E−11	3.12E−10
ko05134	Legionellosis	12	2.37E−07	1.26E−07
ko04512	ECM-receptor interaction	14	1.74E−06	2.42E−06

### Morphological and proliferative differences in rat and mouse chondrocytes

**Fig. 3:**
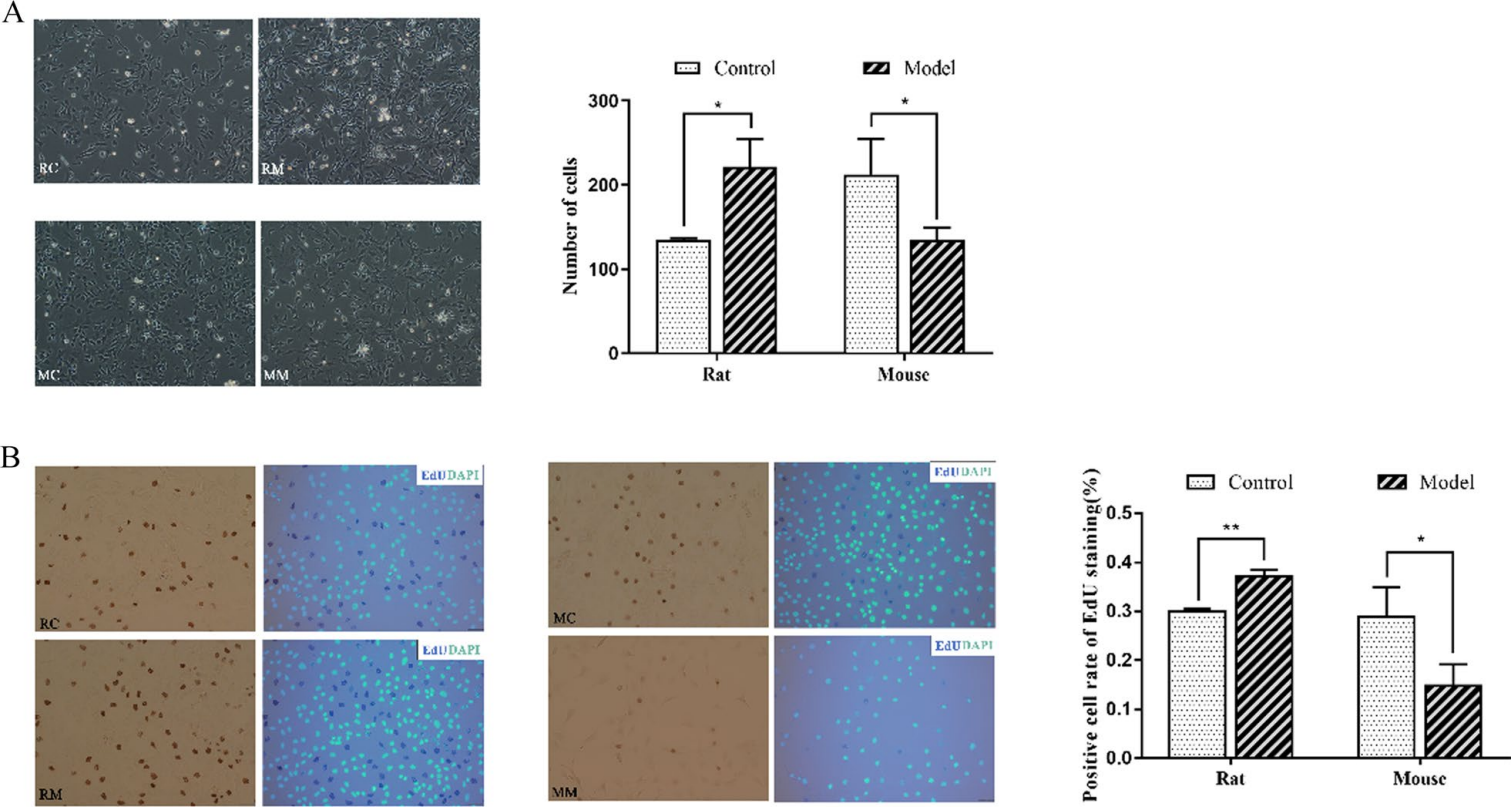
Morphology and proliferation of chondrocytes. **A** Proliferative and morphological changes of chondrocytes stimulated by IL-1β. **B** The effects of IL-1β on the proliferation of chondrocytes were detected by EdU staining. *P < 0.05; **P < 0.001

The results showed that the chondrocytes in the RC and MC groups were polygonal, typical characteristics of chondrocytes. The chondrocytes in the RM and MM groups revealed degenerative and long fusiform changes. The cell density in the RM group was significantly higher than that in the RC group. In contrast, the cell density in the MM group was dramatically lower than that in the MC group (Fig. 3A).

EdU staining showed that the number and rate of EdU-positive cells in the RM group were significantly higher than those in the RC group. In mouse chondrocytes, EdU staining presented conflicting results, e.g. the number and rate of EdU-positive cells in the MM group were significantly lower than those in the MC group (Fig. 3B).

### Differentially expressed genes (DEGs) in RNA-Seq data of rat and mouse chondrocytes

To investigate the differences in gene expression profiles that regulate chondrocyte proliferation, we analysed the RNA-seq results of rat and mouse chondrocytes.

**Fig. 4:**
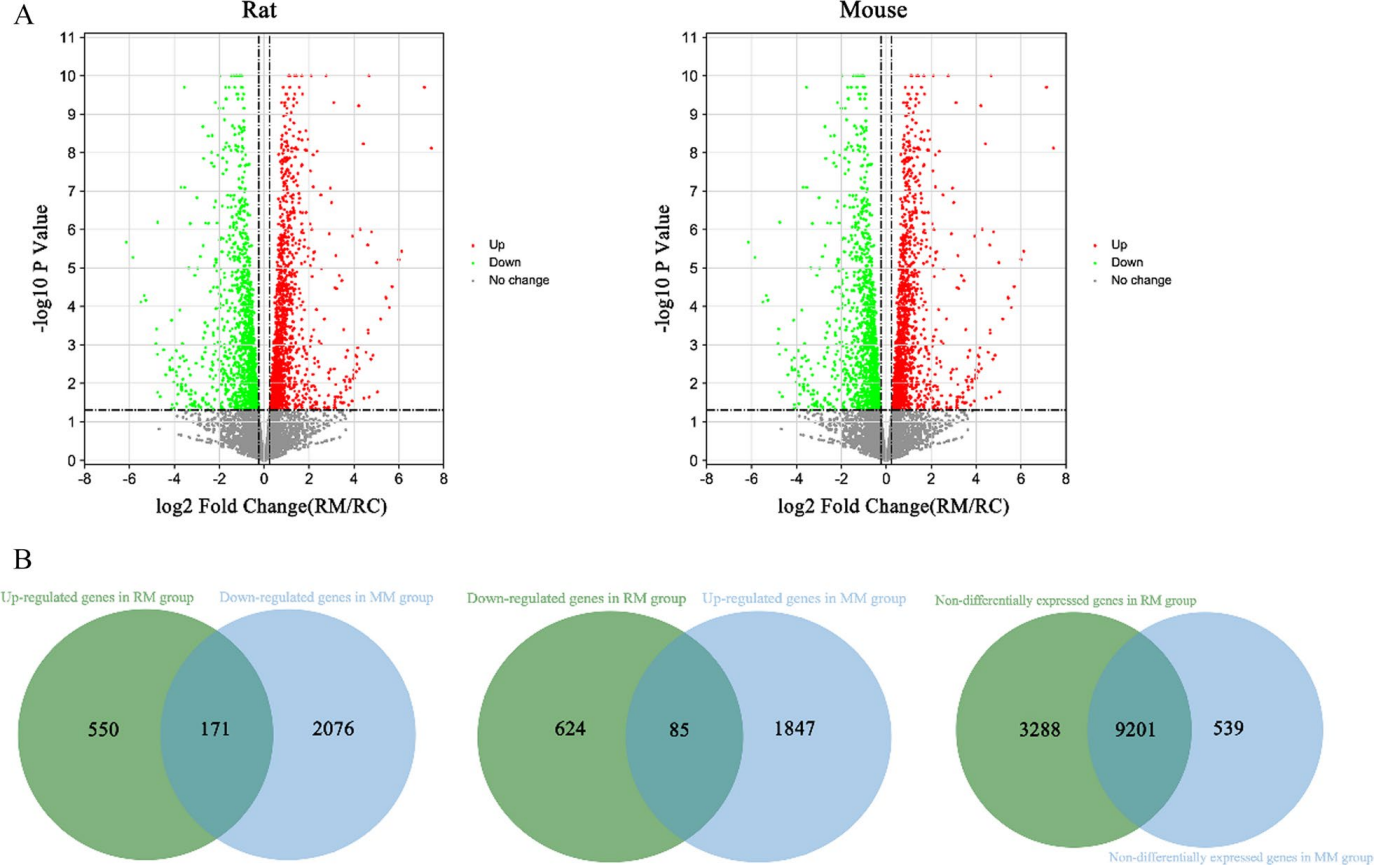
Volcano plots and Venn diagrams of DEGs. **A** Identification of DEGs between the model groups and control groups. *X*-axis represents the log2 transformed fold change. *Y*-axis represents the − log10 transformed significance. Genes with upregulated expression are annotated by red points. Genes with downregulated expression are represented by green points. Grey points represented non-DEGs. **B** Venn diagrams of DEGs between model group

First, the volcanic map showed the differences of 13,919 genes in mRNA expression between the model group and control group in each species (Fig. 4A). Through statistical analysis, this study found 4083 genes with inconsistent expression in the RM and MM groups. Among them, 171 genes had significantly upregulated expression in the RM group and downregulated expression in the MM group. Additionally, 85 genes had significantly downregulated expression in the RM group and upregulated expression in the MM group. In addition, 3288 genes without a significant difference in the RM group had a significant difference in the MM group, whereas 539 genes had no significant difference in the MM group but had a significant difference in the RM group. Therefore, 4083 DEGs were found in rats and mice (Fig. 4B; Additional file 2: Table S2).

### GO and KEGG pathway enrichment analysis of differentially expressed genes (DEGs)

**Fig. 5:**
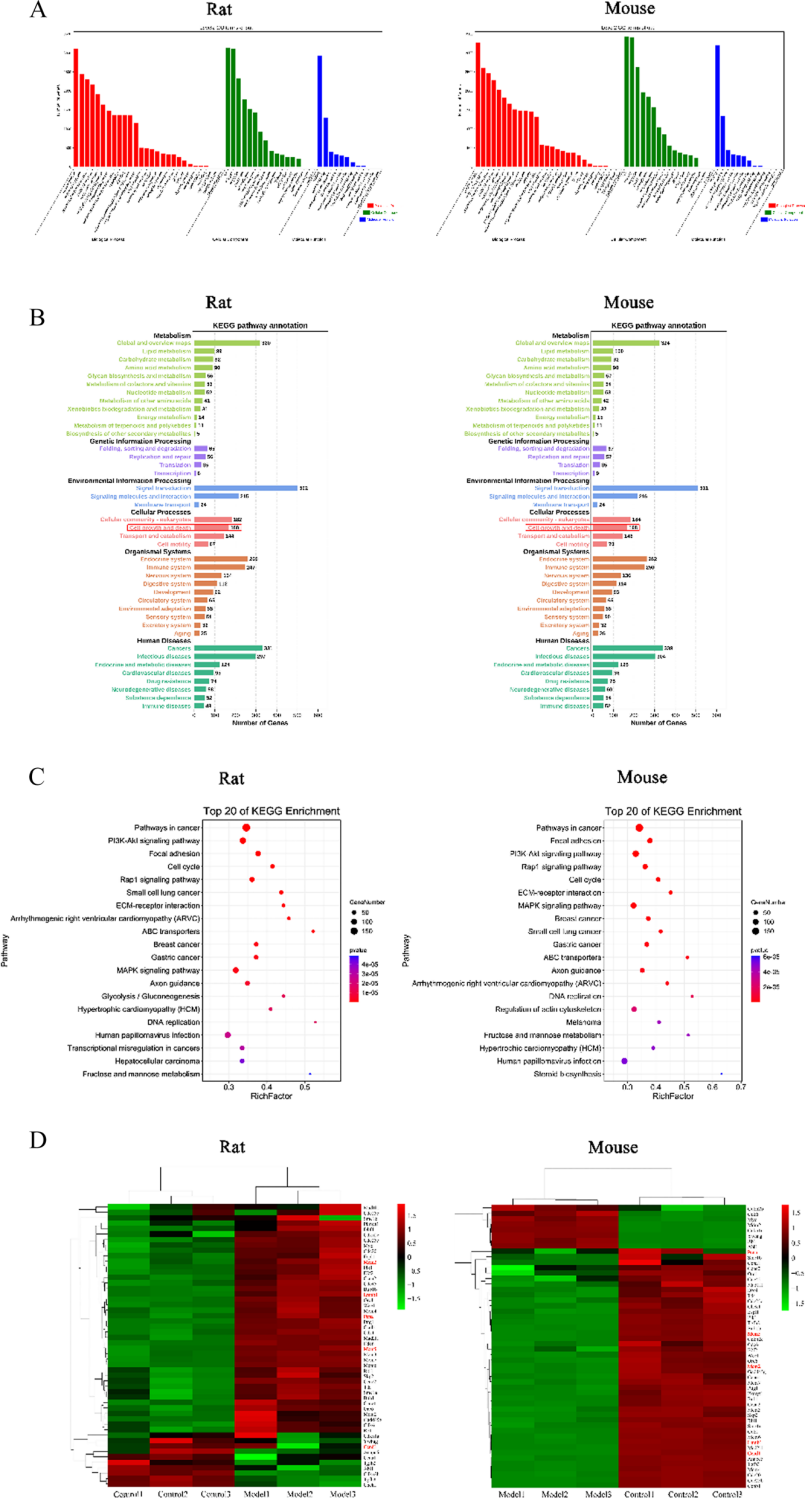
GO and KEGG pathway classification of DEGs. **A** Gene Ontology (GO) classification: red histograms are classified as biological process; green histograms are classified as cellular component and blue histograms are classified as molecular function. **B** Bar chart of KEGG pathway classification. **C** Bubble chart of KEGG pathway functional enrichment. **D** The expression levels of DEGs are represented by the colour from red (high) to green (low)

The differences in the proliferation of the RM and MM groups may be related to these DEGs. As such, these DEGs in rats and mice were further analysed through GO and KEGG pathway enrichment analysis. GO functional classification was conducted to explore the function of annotated genes. In rats and mice, the terms “cellular process”, “biological regulation” and “regulation of biological process”; “cell”, “cell part”, and “organelle”; “binding”, “catalytic activity” and “molecular function regulator” were the most representative of biological process, cellular component and molecular function, respectively (Fig. 5A).

The significant pathways of the DEGs were established by KEGG pathway enrichment analysis. Based on the classification results, 168 DEGs between rats and mice belonged to the category of "cell growth and death" in the category of "cell process"; the signalling pathways in this category were closely related to cell proliferation (Fig. 5B). Among the top five signalling pathways in rats and mice according to the P value, only the "cell cycle" belonged to this category (Fig. 5C; Table 2). The 52 DEGs belonging to the "cell cycle" signalling pathway in rats and mice are shown by hierarchical clustering of a heatmap and include MCM2, MCM5, Lamin B1, PCNA, and Cyclin D1. (Fig. 5D; Additional file 3: Table S3).

**Table 2:** Classification of related pathways (sorted by *P* value)

A class	B class	Pathway name
Human diseases	Cancers	Pathways in cancer
Environmental information processing	Signal transduction	PI3K-Akt signalling pathway
Cellular processes	Cellular community-eukaryotes	Focal adhesion
Environmental information processing	Signal transduction	Rap1 signalling pathway
Cellular processes	Cell growth and death	Cell cycle

### Validation of proliferation-related protein expression in chondrocytes

**Fig. 6:**
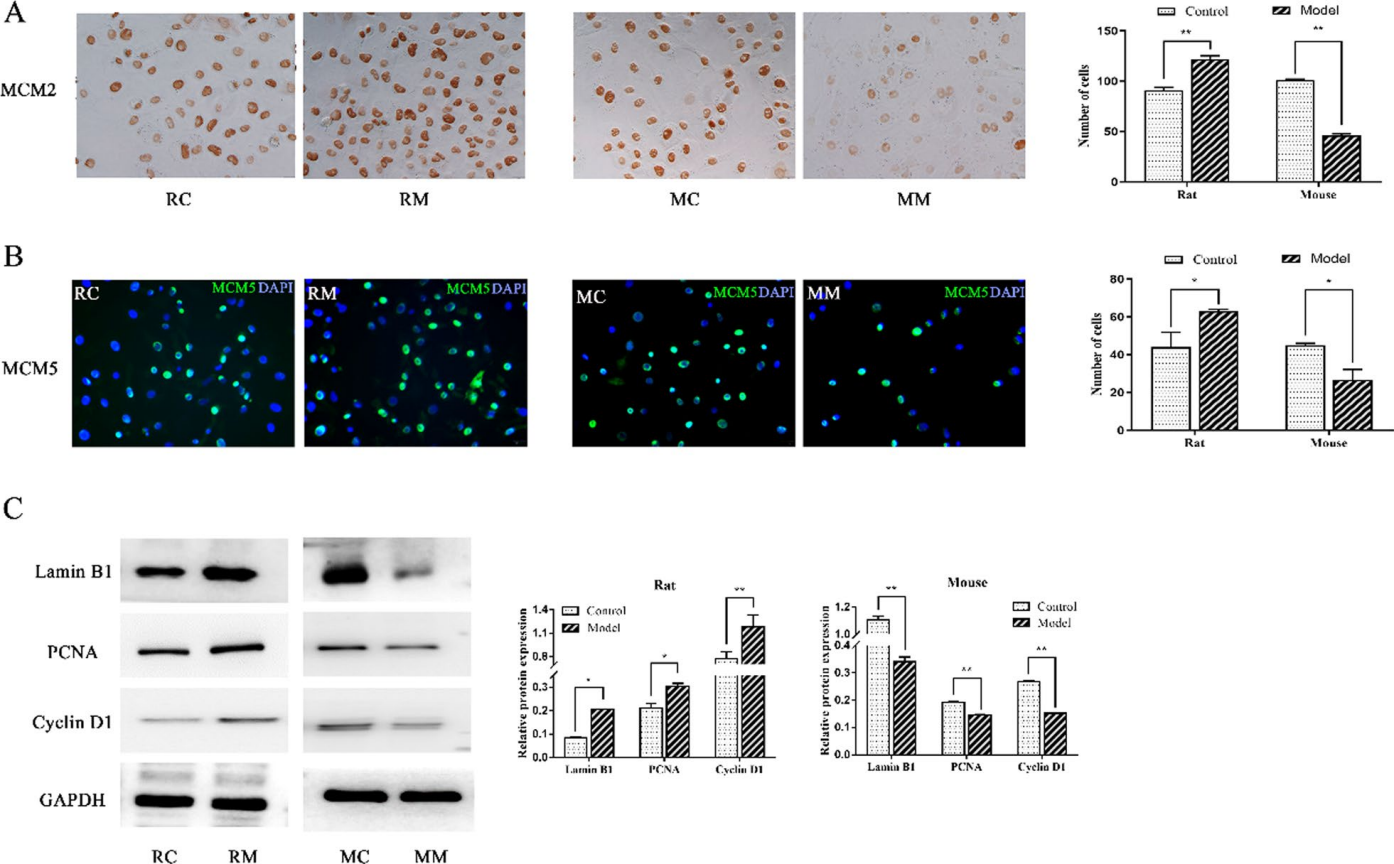
Proliferation-related DEGs in the RM group and MM group. **A** The expression level of MCM2 was detected by immunocytochemistry in rat and mouse chondrocytes after IL-1β intervention. **B** In situ expression of MCM5 was evaluated by cell immunofluorescence (green). The nuclei were counterstained by DAPI (blue). **C** Lamin B1, PCNA and Cyclin D1 expression levels were assessed by Western blots. GAPDH was used as the internal control. *P < 0.05; **P < 0.001

The above five DEGs highly correlated with cell proliferation were selected for analysis to validate the RNA-seq results. The expression levels of MCM2 and MCM5 were detected via immunocytochemistry or immunofluorescence, respectively. The results revealed that, unlike their respective controls, the expression levels of these two proteins were significantly upregulated in the RM group but remarkably downregulated in the MM group (Fig. 6A, B).

As shown in Fig. 6C, IL-1β downregulated the expression levels of Lamin B1, PCNA, and Cyclin D1 in mouse chondrocytes, while upregulating the expression levels of Lamin B1, PCNA, and Cyclin D1 in rat chondrocytes.

## Discussion

Primary chondrocytes isolated from rat or mouse articular cartilage are often used as research objects in experiments in vitro. Therefore, to accurately construct in vitro inflammation models for the study of early OA, we need to explore the differences between in vitro inflammation models of rat and mouse chondrocytes.

This study found that the expression patterns of related cytokines, chemokines, matrix metalloproteinases, and collagen were consistent between rat and mouse chondrocyte inflammatory models after RNA-seq. Notably, genes are biological markers that directly reflect the biosynthesis and catabolism of chondrocytes in OA progression. For instance, interleukins are inflammatory cytokines, and IL-1, IL-6, and IL-18 are proinflammatory factors [16, 17], while IL-13 is an anti-inflammatory factor [18]. Additionally, CXCL1, CXCL2, CXCL3, and CXCL16 belong to the CXC chemokine subfamily [19]. Inflammatory cytokines and chemokines are vital molecules in OA pathogenesis. In the chondrocytes of patients with OA, the content of these cytokines increased to varying degrees; therefore, their expression changes are used as a reference index to establish the inflammatory state in OA development [20–22].

MMP-3 and MMP-13 are matrix metalloproteinases (MMPs); they are the primary collagenases for cartilage degradation and are closely related to the pathological changes of OA [23, 24]. Type I collagen is a marker of fibrocartilage and exists throughout the development of OA; type II collagen is the most important collagen in articular cartilage and supports articular cartilage tissue. Moreover, type IX collagen plays a crucial role in the tissue and stability of the cartilage extracellular matrix [25–27]. By analysing RNA-seq results, all these genes, including inflammatory factors, chemokines, matrix metalloproteinases, and collagen, revealed a similar expression trend in the presence of IL-1β. Therefore, based on the biosynthesis and metabolism of cartilage in OA progression, no difference was noted between the inflammatory model of rat chondrocytes and mouse chondrocytes.

Rat and mouse chondrocyte inflammatory models have similar manifestations in the expression trend of the genes described above under similar conditions, including cell seeding density, IL-1β concentration, and shorter intervention time. However, rat chondrocytes showed significant increases, and mouse chondrocytes showed significant decreases.

To explore the reason, we further analysed and mined the sequencing results. Consequently, 52 genes were closely related to proliferation. Based on the functional GO categorizations, these genes are mainly implicated in cell metabolism. Furthermore, these genes were primarily concentrated in the "cell cycle" signal pathway belonging to "cell growth and death" via KEGG pathway enrichment analysis.

The related genes involved in the regulation of the "cell cycle" also directly regulate the proliferation of chondrocytes in rats and mice, as the genes included MCM2, MCM5, PCNA, Cyclin D1 and Lamin B1 [28–32]. The protein expression levels of MCM2, MCM5, Lamin B1, PCNA, and Cyclin D1 were significantly upregulated in the RM group and remarkably downregulated in the MM group. These findings further verify the RNA-seq results. Our results show that in a short time, a similar IL-1β concentration had the opposite effect on the proliferation of rat and mouse articular chondrocytes. That is, the same inflammatory stimulation conditions promoted the proliferation of rat chondrocytes in a short time period while inhibiting the proliferation of mouse chondrocytes.

Previous studies used IL-1β to stimulate rat chondrocytes and observed their proliferation. The proliferation of rat chondrocytes was significantly inhibited [33, 34]. These findings conflicted with our results. To explore the underlying mechanism, we passaged the chondrocytes used in these studies numerous times or cultured them in vitro for a prolonged period. Chondrocytes may be dedifferentiated, losing their proliferative capacity. Based on OA samples from surgery, chondrocytes from OA cartilage are usually in the middle and late stages of OA [35]. At this stage, the catabolism of chondrocytes is accelerated, and chondrocytes are prone to cellular senescence after culturing in vitro.

Nevertheless, in the early stage of OA, the local loss of cartilage surface proteoglycan and the cleavage of type II collagen trigger a transient increase in chondrocyte proliferation and metabolic activity [36, 37]; unlike healthy chondrocytes, OA chondrocytes have a higher cell proliferation rate [38–40]. Moreover, proliferated human chondrocytes show fibroblast-like morphology and synthesize little extracellular matrix [41]. Therefore, based on the above results of chondrocyte proliferation, extracellular matrix synthesis, and chondrocyte morphology, the rat chondrocyte inflammation model is more suitable to simulate the cartilage inflammation state in the early stage of OA than the mouse chondrocyte inflammation model. In addition, the corresponding research findings are more reliable.

This study has compelling limitations. First, we only explored and compared in vitro inflammatory models of rat and mouse chondrocytes, yet they have not been simultaneously compared with inflammatory models of human chondrocytes. As such, based on the available relevant findings, we can only speculate that the rat chondrocyte inflammation model is more suitable for early OA-related studies. In future research, we should establish additional species of early chondrocyte inflammation models and compare them with human chondrocytes in early OA. This analysis will provide more reliable inflammation models for early OA in vitro experiments.

## Conclusions

In conclusion, the expression of cytokines, chemokines, matrix metalloproteinases, and collagen in rat and mouse chondrocyte inflammation models was similar under similar inflammatory stimulation for a short period of time. However, considerable differences were noted in cell proliferation. Therefore, based on our results, the rat chondrocyte inflammation model may help in the study of the early pathological mechanism of OA.

## Supplementary Information

**Additional file 1.** A list of 635 genes with similar expression patterns in rats and mice.

**Additional file 2.** A list of 4083 differentially expressed genes in rats and mice.

**Additional file 3.** A list of 52 differential expressed genes belonging to the “cell cycle” signaling pathway.

**Additional file 4.** Number of published reports on cell models of osteoarthritis.

## Data Availability

The datasets used and/or analysed during the current study are available from the corresponding author on reasonable request.

## Abbreviations

OA: Osteoarthritis
MCM2: Minichromosome maintenance 2
MCM5: Minichromosome maintenance 5
PCNA: Proliferating cell nuclear antigen
RC: Rat control group
MC: Mouse control group
RM: Rat model group
MM: Mouse model group
RIN: RNA integrity number
FPKM: Fragments per kilobase million
DEG: Differentially expressed gene
EdU: 2.4 5‑Ethynyl‑2′‑deoxyuridine
PBS: Phosphate-buffered saline
DAB: Diaminobenzidine
MMPs: Matrix metalloproteinases
