# Supplementary figures and images for: Similarities and differences between rat and mouse chondrocyte gene expression induced by IL-1β
Source: J Orthop Surg Res. 2022 Feb 4;17:70. doi: 10.1186/s13018-021-02889-2 (PMC8815127; doi:10.1186/s13018-021-02889-2)

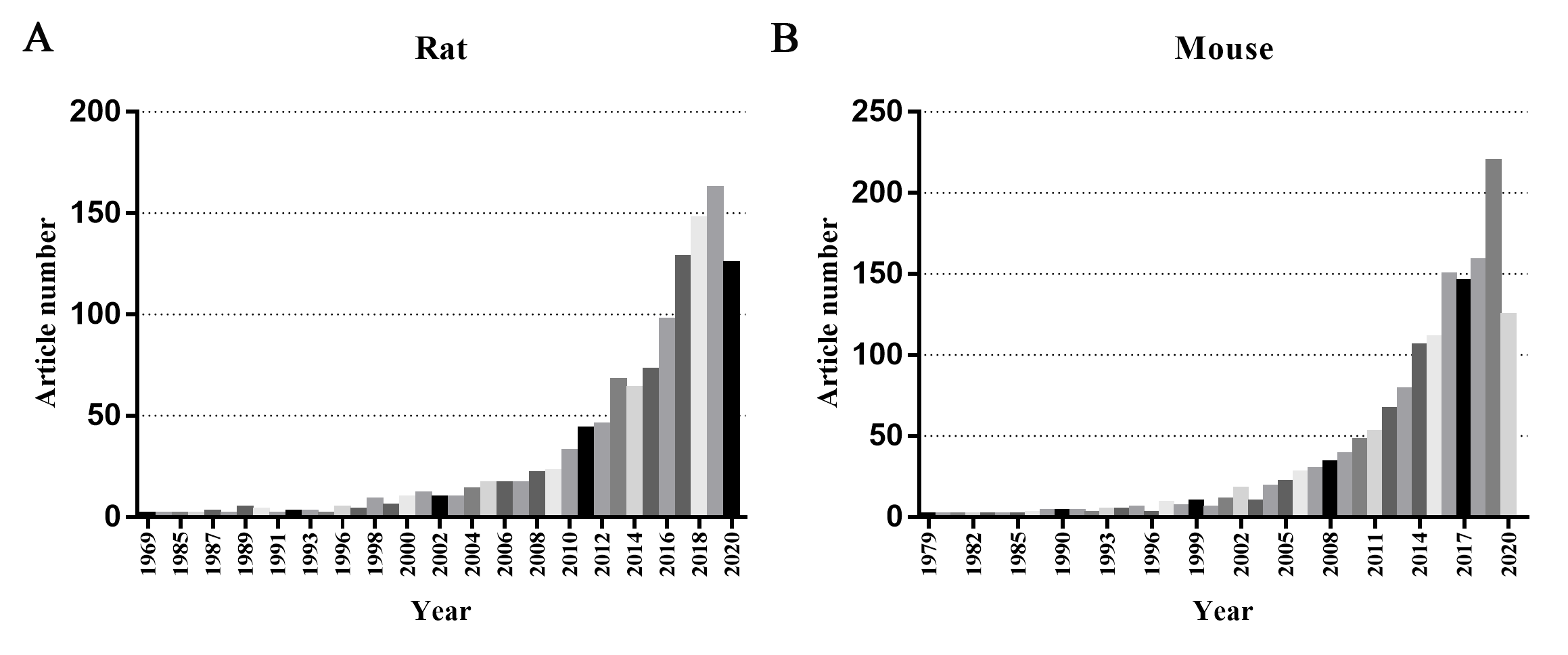

Supplement: Supplementary file 4 — Additional file 4. Number of published reports on cell models of osteoarthritis. [file 13018_2021_2889_MOESM4_ESM.tif]
